# Supplementary material for: Visual feedback manipulation in virtual reality to influence pain-free range of motion. Are people with non-specific neck pain who are fearful of movement more susceptible?
Source: PLoS One. 2023 Jul 5;18(7):e0287907. doi: 10.1371/journal.pone.0287907 (PMC10321611; doi:10.1371/journal.pone.0287907)
Supplement: S1 Appendix — (DOCX) [file pone.0287907.s001.docx]

**Supporting information**

**S1. Appendix: Pilot study ‘Creating illusions: Perception of Gain Changes when Manipulating the Visual Feedback with Virtual Reality’**

**Introduction:**

Virtual Reality (VR) offers possibilities for influencing pain perceptions. By manipulating the visual feedback in a VR environment, you can create the illusion of moving more or less than you actually do, which influences movement evoked pain [1]. To create such an illusion, a technique called ‘redirected walking’ can be used. The factor that translates real neck rotation to virtual rotation is called the ‘gain’ [2]. It is stated that, within certain limits, this manipulation goes unnoticed [1, 2]. This study was aimed to determine the spectrum of rotation gains that were more likely to be judged as ‘not manipulated’ than ‘manipulated’.

**Method:**

*Participants and equipment*

A cross-sectional study was conducted with healthy participants, aged between 18 and 65. Participants were excluded if they had severely impaired vision. Participants were wearing the VR-headset (Oculus Rift: Oculus VR, Irvine, CA) while sitting on a chair with their trunk fixated in order to prevent them from moving their torso (see Fig 1 in the main study). The VR-headset was connected with a Windows running computer, where 4 life-realistic virtual environments (i.e. a forest, a loft, a workman’s shed and an undulating landscape) were saved and functioned with the programme Unity 5.3.1. (Unity Technologies, San Francisco, CA, USA).

*Procedure*

Participants were asked to look around (i.e. rotate their head to the right, then to the left and back to the middle) in 4 different VR environments that were successively projected into the VR-headset. They were told beforehand that the visual feedback was manipulated in some occasions and not in others. In each measurement a different gain condition, ranging from 0.4 gain to 1.6 gain, was presented. Each of the 11 rotation gains (0.4, 0.5, 0.6, 0.7, 0.8, 1.0 (=no manipulation), 1.2, 1.3, 1.4, 1.5 and 1.6) was presented twice, so 22 measurements were obtained from each participant. For each repetition, the participant was instructed to rotate the head to the right and left without a pause and then report the researcher whether the visual feedback was manipulated or not. In case the participant thought that the visual feedback was manipulated, he/she was asked if the feedback overstated or understated true rotation. The outcome measures of the pilot study were 0= no manipulation, 1= yes, the visual feedback overstated true rotation (i.e. faster) and 2= yes, the visual feedback understated true rotation (i.e. slower).

Prior to the initial testing, four different standardized presets were created to determine the order of the different gain conditions. In each preset a gradual descending or ascending order was followed in order to avoid large gain changes, which would reveal the manipulation more evidently. Seven or eight participants were randomly assigned to follow one of the four presets. Repetitions were delivered in 3 blocks of 7-8 measurements. A pause of two minutes, in which the participant was asked to close his/her eyes while they kept wearing the VR-headset, was needed to load the new gains and also to prevent participants from motion sickness.

Data-analysis

The percentage of measurements in which participants thought the visual feedback was ‘manipulated’ or was ‘not manipulated’ was calculated for each of the eleven gains, using frequencies and crosstabs on IBM SPSS Statistics version 23 software. This was expressed in a graph. In the graph it could be seen in which spectrum the rotation gains were more likely to be judged as ‘not manipulated’ than ‘manipulated’.

**Results:**

30 healthy people participated (8 men; mean age = 30 years, SD= 10.6, Mdn=26). The results showed us that the gain conditions between 0.7 and 1.4 were judged more often as ‘not manipulated’ than ‘manipulated’.

**S1 Fig.** Results pilot study: The percentage of rotation measurements in which the participant thought the visual feedback was manipulated (faster or slower) or was not manipulated in different rotation gains.

**S1 Table.** Results pilot study: Perception of visual feedback manipulation

| Percentage of rotations noticed as… | Gain condition | | | | | | | | | | |
| --- | --- | --- | --- | --- | --- | --- | --- | --- | --- | --- | --- |
|  | **0,4** | **0,5** | **0,6** | **0,7** | **0,8** | **1** | **1,2** | **1,3** | **1,4** | **1,5** | **1,6** |
| ‘not manipulated’ (%) | 13.3 | 13.3 | 13.3 | 46.7 | 50.7 | 71.1 | 68.7 | 55.0 | 43.3 | 30.0 | 28.3 |
| ‘manipulated; faster than actual rotation’ (%) | 21.7 | 23.3 | 15.0 | 8.3 | 13.4 | 15.8 | 20.9 | 25.0 | 43.3 | 53.3 | 60.0 |
| ‘manipulated; slower than actual rotation’ (%) | 65.0 | 63.3 | 71.7 | 45.0 | 35.8 | 13.2 | 10.4 | 20.0 | 13.3 | 16.7 | 11.7 |

**Discussion:**

The aim of this pilot study was to present the spectrum of rotation gains within VR that were more likely to be judged as ‘not manipulated’ than ‘manipulated’, based on the answers of healthy people who were asked to look around in 4 presented VR environments in which the gain differed. It was hypothesized that this spectrum was at least between 0.8 and 1.2, but might be larger (0.8-1.5) [1, 2]. The results showed that most people didn’t notice the manipulation of the visual feedback between in the gain conditions ranging from 0.7 to 1.4. This spectrum is wider than the utmost gains Harvie found in his pilot study [1]. An explanation for this difference could be that the quality of VR has increased enormously in recent years, making the VR-images less distorted when manipulating the feedback and therefore remain more realistic. Besides, Harvie used only 9 participants for his pilot study, so these results may be coincidental. The results found in the current study were consistent with the study of Steinicke regarding the fact that it is more difficult to detect the manipulation in an overstated conditions (so it seems like you are moving more than you actually do) than in understated conditions (it seems like you are moving less than you actually do) [2].

In this study participants were informed beforehand about the manipulation of the visual feedback. Therefore participants were focused on this issue. It is expected that the spectrum in which the manipulation is not noticed would even be wider if they were not informed prior to the experiment. A limitation of the current study was that only 4 VR environments were used, which made it possible for participants to compare multiple conditions, this could have influenced the results. Therefore it is recommended to use a gain between 0.7 and 1.4 and multiple VR environments if virtual reality is used to create an illusion, without being noticed.

**References**

1. Harvie DS, Broecker M, Smith RT, Meulders A, Madden VJ, Moseley GL. Bogus visual feedback alters onset of movement-evoked pain in people with neck pain. Psychol Sci. 2015;26(4):385-92.

2. Steinicke F, Bruder G, Jerald J, Frenz H, Lappe M. Analyses of human sensitivity to redirected walking. Proceedings of the 2008 ACM symposium on Virtual reality software and technology; Bordeaux, France: Association for Computing Machinery; 2008. p. 149–56.
