# Supplementary material for: Visual feedback manipulation in virtual reality to influence pain-free range of motion. Are people with non-specific neck pain who are fearful of movement more susceptible?
Source: PLoS One. 2023 Jul 5;18(7):e0287907. doi: 10.1371/journal.pone.0287907 (PMC10321611; doi:10.1371/journal.pone.0287907)
Supplement: S2 Appendix — (DOCX) [file pone.0287907.s002.docx]

**S3. Appendix: Results of the data-analyses using the absolute data (Range of Rotation in degrees)**

*Statistical assumptions*

Based on visual inspection of the Q-Q plots, the histograms and the Kolmororov-Smirnov test (all p>0.154), the absolute data were considered sufficiently normally distributed. For the analysis based on the TSK score, Levene’s test revealed that there were no violations of the assumption of homogeneity of variance between the gain conditions. However, when the FABQ_pa_ score was used, variances differed between the gain conditions. Sphericity was violated for both analyses and the Greenhouse-Geisser EPSILON was <0.75, therefore the Greenhouse-Geisser correction was used in both analyses [1].

*Effect of fear and visual feedback manipulation on cervical pain-free range of motion*

Using the absolute data, the ANOVA revealed that the interaction effect between kinesiophobia (as determined by the TSK; p=0.116, ղ_p_^2^=0.037) or fear of physical activity (as determined by the FABQ_pa_; p=0.129, ղ_p_^2^=0.035) and visual feedback manipulation on cervical pain-free range of motion was small and not significant. This indicates that the cervical pain-free range of motion when visual feedback understates or overstates true neck rotation was not depending on the presence of fear (see S3 Fig).

Further analyses showed a main effect of gain on cervical pain-free neck range of motion (TSK: p=0.001, ղ_p_^2^=0.134; FABQ_pa_: p<0.001, ղ_p_^2^=0.159). Contrasts revealed that this effect was large for the overstated condition, compared to the control condition (TSK: p<0.001, ղ_p_^2^=0.187; FABQ_pa_: p<0.001, ղ_p_^2^=0.276), while not present for the understated condition (TSK: p<0.303, ղ_p_^2^=0.017; FABQ_pa_: p=0.743, ղ_p_^2^=0.002), indicating that the cervical pain-free range of motion decreases when visual feedback overstates true rotation. The absolute range of motion scores demonstrate that the mean difference between the understated condition (0.7 gain) and the overstated condition (1.3 gain) was larger for people with kinesiophobia (∆ ROM of 7.3 degrees (95%CI: 1.8, 12.7) and with fear of physical activity (∆ ROM of 7.5 degrees (95%CI: 2.2, 12.7) than in people without fear of movement (∆ ROM of 2.6 (95%CI: -0.8, 5.9), while their total cervical pain-free range of motion was noticeably smaller (see Table 2). These mean changes in the fear groups might be clinically interesting, as a difference above the value of 6.5 degrees is considered a ‘true difference’ [2].

Between subject tests showed a main effect of kinesiophobia on mean cervical pain-free range of motion and this was a medium to large effect (TSK: p<0.001, ղ_p_^2^=0.219), FABQ_pa_: p=0.010, ղ_p_^2^=0.102)). The mean (SD) range of rotation in the control condition in people with kinesiophobia (TSK) was 96.5 (32.4) degrees, in the fear of physical activity group 106.9 (38.4) and in the ‘no fear’ group 128.5 (26.6) degrees (for further specification see Table S3).

**Table S3.** Influence of visual feedback manipulation on cervical pain-free range of motion

| Gain condition | Total range of motion (degrees) (mean [95%CI]) | | | |
| --- | --- | --- | --- | --- |
|  | **All participants**  (N=75) | **No fear of movement**  (TSK≤37 & FABQ_pa_≤14)  (N=46^*/**^) | **Kinesiophobia**  (TSK>37)  N=19^*^ | **Fear of physical** **activity**  (FABQ_pa_>14)  N=18^**^ |
| Absolute data^1^ |  |  |  |  |
| 0.7 gain  1.0 gain  1.3 gain | 120.8 [114.0, 127.5]  120.9 [113.7, 128.1]  117.0 [109.5, 124.4] | 127.3 [120.1, 134.4]  128.5 [121.1, 135.9]  124.7 [116.9, 132.5] | 99.5 [84.4, 114.6]  96.5 [80.8, 112.1]  92.3 [76.6, 107.9] | 108.7 [91.0, 126.4]  106.9 [87.8, 126.0]  101.2 [81.6, 120.8] |

^1^ Absolute data: the total cervical range of motion (i.e., the sum of left and right rotation) in degrees * For one participant, data regarding the Tampa score was missing. Therefore, this participant was not included in one of the subgroups. **For two participants, the FABQ_pa_ score was missing. Therefore, these participants were not included in one of the subgroups. N: number; 95%CI: 95% Confidence Intervals [lower bound, upper bound]

**References**

1. Field A. Repeated-Measures Designs. Discovering Statistics Using IBM SPSS Statistics. Fifth ed. Los Angeles, London, New Dehli, Singapore, Washington DC, Melbourne: SAGE; 2018. p. 651-702.

2. Audette I, Dumas JP, Cote JN, De Serres SJ. Validity and between-day reliability of the cervical range of motion (CROM) device. J Orthop Sports Phys Ther. 2010;40(5):318-23.

**S3 FIGURE**

**A**


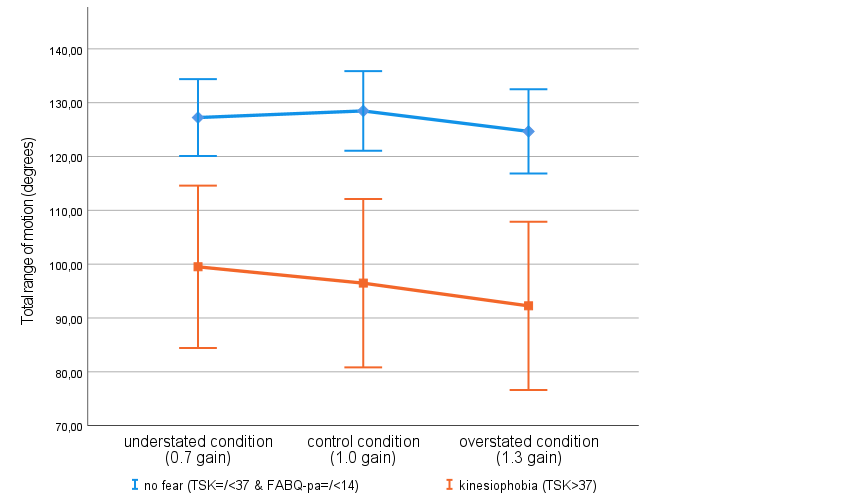


**B**


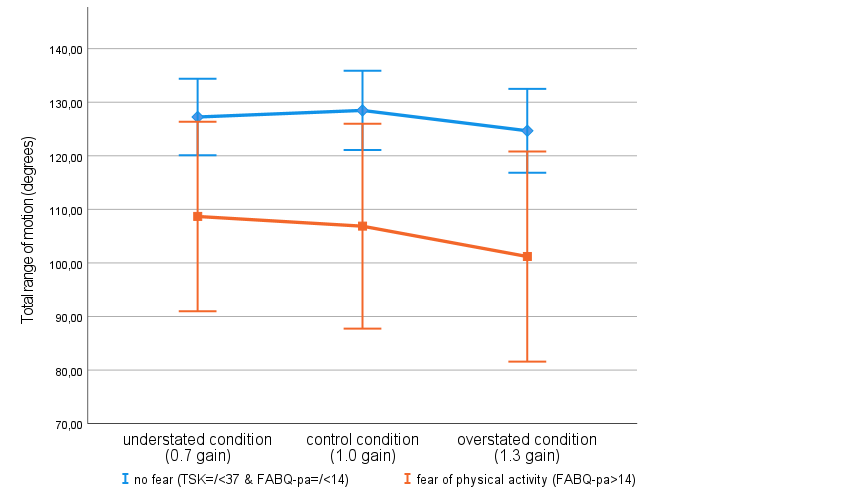


**S3 Fig.** Effect of visual feedback manipulation on cervical pain-free range of motion in people without fear of movement (Tampa≤37 & FABQ_pa_≤14) versus people with kinesiophobia (Tampa>37) (S3 Fig. A) and versus people with fear of physical activity (FABQ_pa_>14) (S3 Fig 3B). Please note that the total cervical range of motion is the sum of left and right rotation. The error bars represent the 95% confidence intervals.
