# Supplementary material for: Visual feedback manipulation in virtual reality to influence pain-free range of motion. Are people with non-specific neck pain who are fearful of movement more susceptible?
Source: PLoS One. 2023 Jul 5;18(7):e0287907. doi: 10.1371/journal.pone.0287907 (PMC10321611; doi:10.1371/journal.pone.0287907)
Supplement: S1 Fig — (DOCX) [file pone.0287907.s003.docx]

**S2 FIGURE**


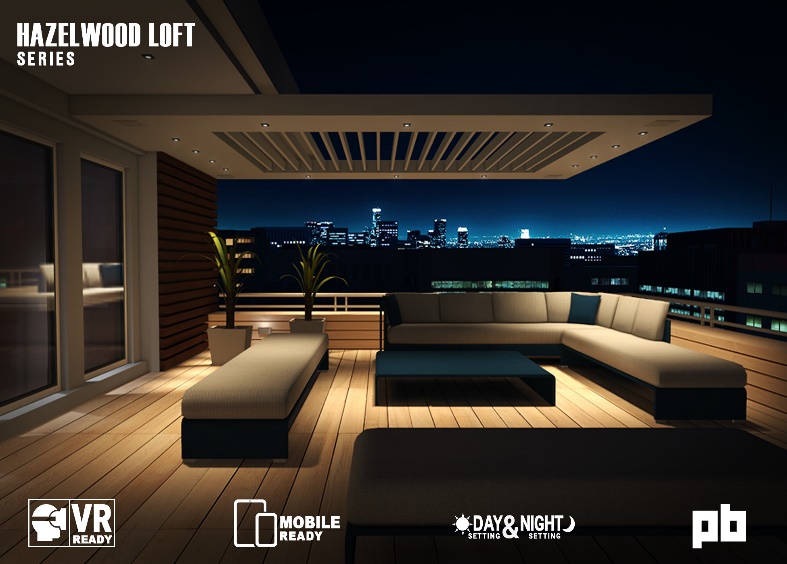

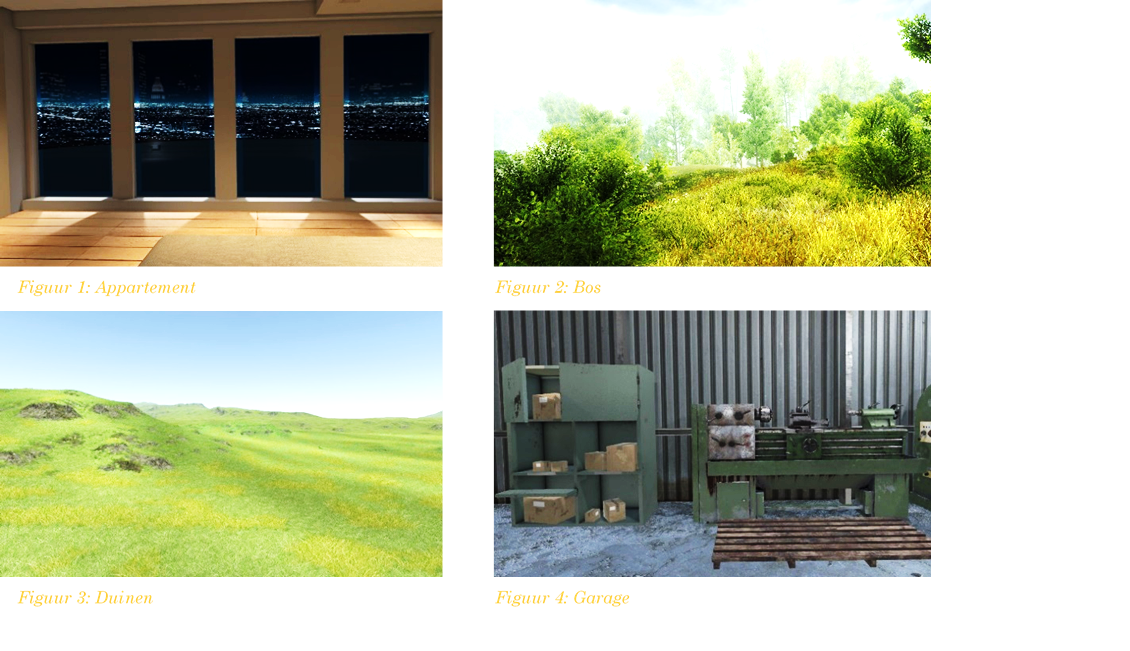


Loft Forest


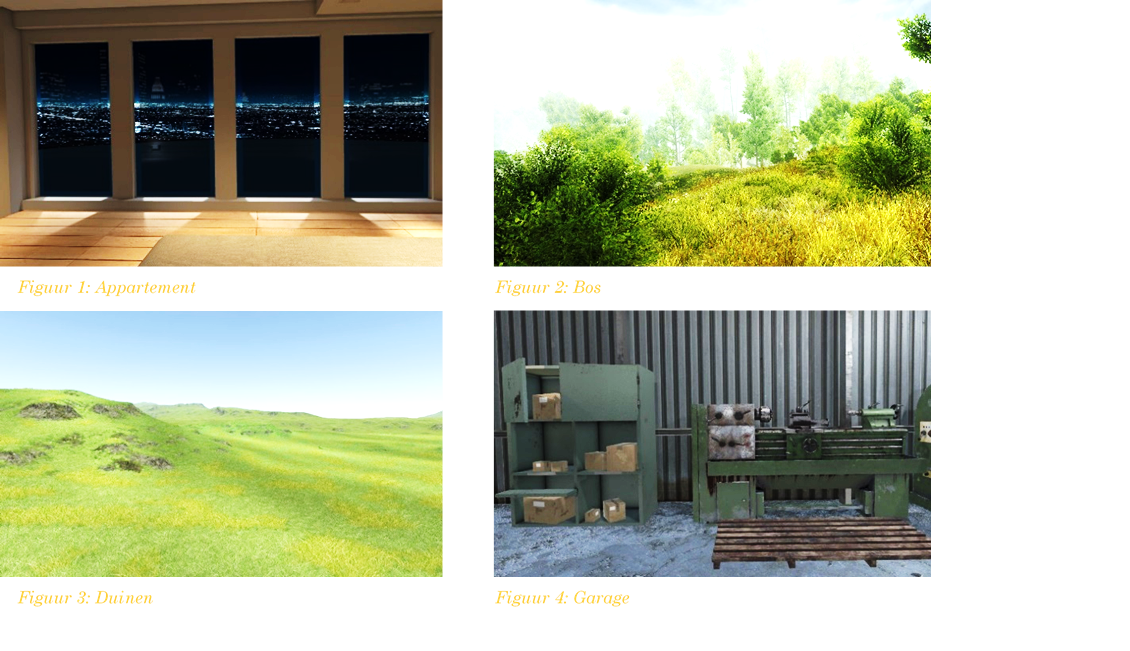

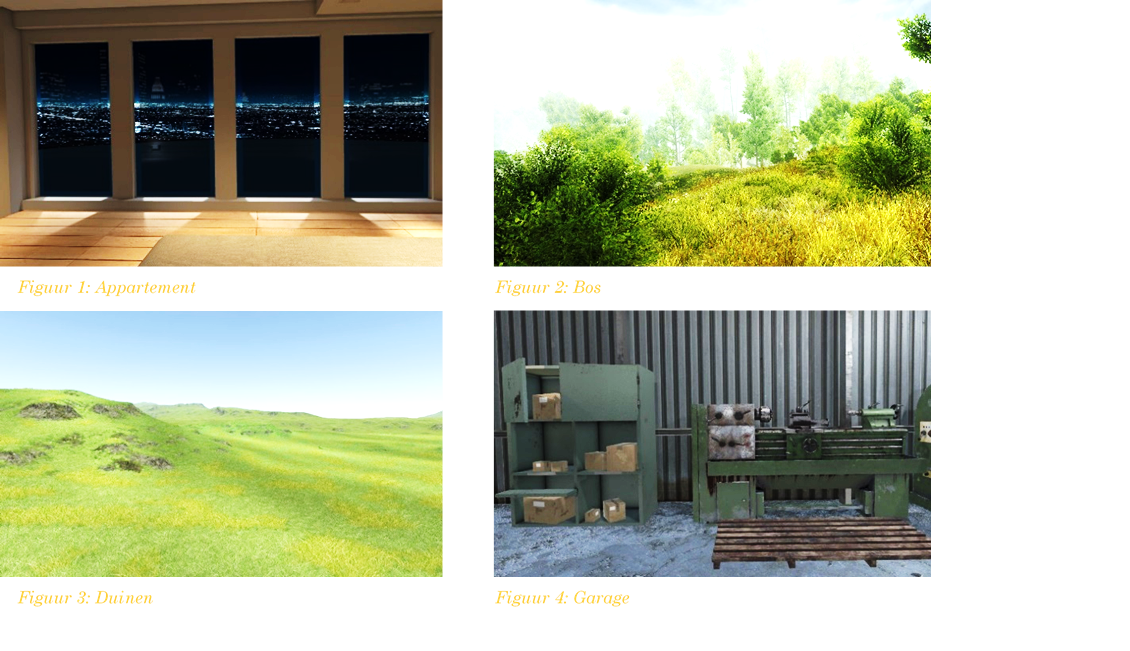


Undulating landscape Workman’s shed


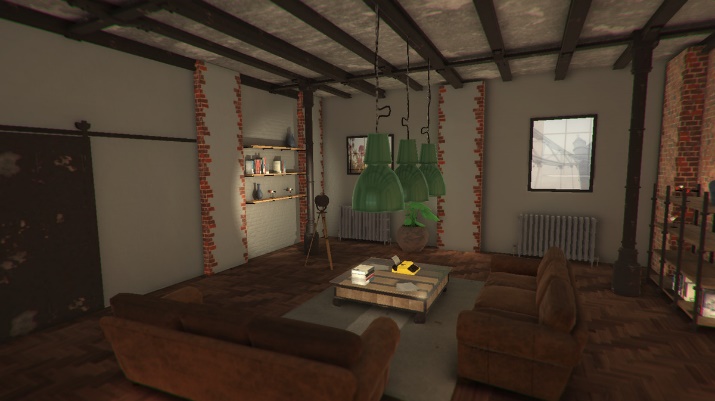

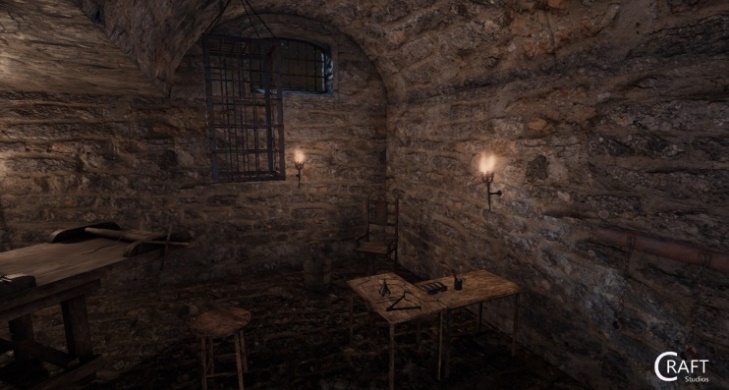


Living room Dungeon

**S2 Fig.** Examples of the Virtual Reality Environments projected in the VR-headset
