# Supplementary material for: Visual feedback manipulation in virtual reality to influence pain-free range of motion. Are people with non-specific neck pain who are fearful of movement more susceptible?
Source: PLoS One. 2023 Jul 5;18(7):e0287907. doi: 10.1371/journal.pone.0287907 (PMC10321611; doi:10.1371/journal.pone.0287907)
Supplement: S1 Table — (DOCX) [file pone.0287907.s004.docx]

**Table S4.** Exploratory analysis: Influence of visual feedback manipulation on cervical pain-free range of motion in people who scored positive or negative on both scales

| Gain condition | All participants  (N=56) | No fear of movement  (TSK≤37 & FABQ_pa_≤14)  (N=46^*/**^) | Fear of movement  (TSK>37 & FABQ_pa_>14)  (N=10^*/**^) |
| --- | --- | --- | --- |
| Relative data^1^ |  |  |  |
| 0.7 gain  1.0 gain  1.3 gain | 1.008 [0.991, 1.025]  1.0 [1.0, 1.0]  0.964 [0.948, 0.981] | 0.993 [0.978, 1.009]  1.0 [1.0, 1.0]  0.969 [0.951, 0.987] | 1.052 [0.979, 1.124]  1.0 [1.0, 1.0]  0.925 [0.874, 0.976] |
| Absolute data^2^ |  |  |  |
| 0.7 gain  1.0 gain  1.3 gain | 120.8 [114.0, 127.5]  120.9 [113.7, 128.1]  117.0 [109.5, 124.4] | 127.3 [120.1, 134.4]  128.5 [121.1, 135.9]  124.7 [116.9, 132.5] | 89.8 [71.7, 108.0]  86.1 [67.7, 104.6]  80.0 [61.6, 98.5] |

^1^ Relative data: a proportion of the mean cervical range of rotation in the control condition. ^2^Absolute data: the total cervical range of motion (i.e., the sum of left and right rotation) in degrees * For one participant, data regarding the Tampa score was missing. Therefore, this participant was not included in one of the subgroups. **For two participants, the FABQ_pa_ score was missing. Therefore, these participants were not included in one of the subgroups. N: number; 95%CI: 95% Confidence Intervals [lower bound, upper bound]
