# Supplementary material for: Visual feedback manipulation in virtual reality to influence pain-free range of motion. Are people with non-specific neck pain who are fearful of movement more susceptible?
Source: PLoS One. 2023 Jul 5;18(7):e0287907. doi: 10.1371/journal.pone.0287907 (PMC10321611; doi:10.1371/journal.pone.0287907)
Supplement: Attachment — Submitted filename: Response to Reviewers_VR_Fear.docx [file pone.0287907.s006.docx]

| datum | ons kenmerk | uw brief van | uw kenmerk |
| --- | --- | --- | --- |
| 26 April 2023 |  |  |  |
|  |  |  |  |

To:	Prof. Mariella Pazzaglia

Academic Editor PlosOne

Dear Prof. Mariella Pazzaglia,

Thank you for giving us the opportunity to revise our manuscript entitled *‘Visual feedback manipulation in virtual reality to influence pain-free range of motion. Are people with pain who are fearful of movement more susceptible?’* for PlosOne (Article number: PONE-D-22-28987).

We would like to thank you and the reviewers for the helpful comments, positive feedback and clear suggestions for improving the quality of the manuscript. We have addressed each of the points raised. In the response letter, the reviewer comments are printed in black and italic, followed by our response in regular blue font.

All authors read and approved the revised manuscript. We look forward to your response on the revised manuscript.

On behalf of all the authors,

Yours sincerely,

Michel Coppieters

**Accompanying response letter**

**Reviewer # 1**

Comments to the Author
In this paper, the authors study how the manipulation of visual experience through the use of virtual reality can affect the perception of pain due to movement in participants with non-specific neck pain. The main result of the study is to have identified a greater amplitude of pain-free movement when visual manipulation reduces the perceived rotation angle compared to when it is increased. The effect reached significance only within the group without kinesiophobia.

The article is written in appropriate and easily understandable language. Data analysis, the process is fully described and seems to have been conducted appropriately in relation to the characteristics of the research.

An interesting aspect is also the easy transportability in the clinical practice of these types of procedures. However, the literature on these aspects is more extensive than perhaps transpires from what is presented in the introduction and could benefit from slight enrichment.

At the discretion of the authors, I believe that some passages could benefit from brief clarification or elaboration.

*Abstract*

*1. The statements in the abstract should be made more explicit and understandable. In particular, results and discussion could clearly report not only that effects have been identified but also in what direction these are going (i.e. effects on participants’ painless movements ).*
Response: We agree with this comment. We adjusted this in the abstract and reported the direction of the effects (lines 47-50).

*2. Line 56 - The concept of "non-specific neck pain" should be better defined.*
Response: Although this term is widely used, we can imagine that specifying the concept of 'non-specific neck pain' leads to more clarity. Therefore we defined it as ‘neckpain without a specific underlying pathology’^1^ (See line 39). A further specification of the characteristics of the participants is described in the selection criteria in the Methods section (See lines 93-100).

*3. Lines 66-67 and Fig.1A. The authors refer to another study, but a figure in reference (6) depicts the same subjects with the same experimental setup with the same caption as fig 1A, this may be confusing. Please clarify in the text, photo/picture caption, or both if necessary.*

Response: It is correct that we published a separate study using the same materials (e.g., VR headset, chair, fixation belt, computer) in an equivalent setting (as shown in the picture in figure 1A). However, this previously published article focused on different research questions, namely: (1) to determine whether pain-free range of motion increased and decreased when visual feedback understated or overstated true neck rotation with 20%, (2) to explore whether people with (long-lasting) chronic neck pain were more prone to the effect of visual feedback manipulation than people with subacute neck pain. This study did not address the effect of VR in people with fear of movement. So, the research questions from the current study generated from the first study, used different participants, different gain conditions (i.e., manipulation of the visual feedback with 30% rather than 20%) and different VR-environments. We clarified this in the picture (see figure 1). Despite similarities, the pictures differ from the pictures used in the previous publication. Taking into account point 8, we added two examples of the environments used to figure 1, and made this available directly in the manuscript.

*4. The authors in their manuscript provide all the necessary information. However, for the convenience of the reader, it would seem to me to be useful to indicate in the participants section some basic info (which the authors actually include in the first few lines of the results) in order to understand with more immediacy numerosity and demographic characteristics of the participants.*

Response: We added the info as requested in the Methods (see line 91).

*5. Line 91 - Please be a little clearer about the exclusion criterion. Is the problem in vision or possible problems related to wearing an HMD while wearing glasses? Was corrected to normal vision using contact lenses was ok.*
Response: We have adopted the reviewer’s suggestion and added ‘(e.g., people who had poor vision when not wearing their glasses as they could not be worn in the VR condition. The use of contact lenses was not a problem)’ to the description of the exclusion criteria (see lines 99-100)

*6. Genders are differently represented, and in the rest of the manuscript, there is no mention of gender except in the table. Please include explicitly in the text, either in the participant section or in the results and/or discussion, the rationale for this choice and some consideration of the role of this variable in the study.*

Response: We assessed gender to describe the sample. Indeed, in this study genders are differently represented. Considering the fact that neck pain between the ages of 45 and 64 is almost 50% more common in women than in men^2^, we belief that the distribution within this study reflects the population of people with nonspecific neck pain. We added this interpretation to the text (see line 346-347). We did not look at differences between men and women regarding the influence of fear. Due to the limited sample size of the fear group in this study, it was not possible to determine an effect of gender.

*7. Lines 167-170 Appropriate pilot study was used to test the recognition effect of manipulation on specific stimuli. Although in non-identical procedures, visual perspective manipulation via virtual reality is nonetheless present in the literature, and providing one or two insights might be appropriate for the manuscript and useful to the reader.*

Response: We agree with the reviewer that insights regarding visual perspective manipulation via virtual reality is present in the literature. However, to our best knowledge, the sensitivity to rate of change in gains (applied by redirected walking) is studied in other conditions (e.g. upper limb movements, gait), but is not described related to neck movements (apart from the references that were already mentioned in the study). In our previous study the use of a 20% gain did not influence pain free range of motion^3^. We expected that a larger change in gain was needed to induce an effect. However, we did not know if we could enlarge the gain without being noticed. Therefore we performed the pilot study that is referred to.

*8. Line 186- It might be interesting for the reader to have one or two examples of environments (e.g., 1 natural and 1 artificial) available directly in the manuscript from among the supplementary materials.*

Response: Two examples of the environments used are added to Figure 1 (C and D)

*9. Also, about the environments used, it seems to me that the choice of the types of such environments is not discussed except to avoid possible learning effects and memorization of visual references. Two environments are natural and very bright, while four are artificial and dark. The literature addresses these types of differences from numerous perspectives, however, given the short duration of the exposures and the type of task, it may not be of particular relevance. However, it would be appropriate to include in the manuscript, also in the discussion (around lines 344-347 would seem appropriate to me), some brief consideration and reference regarding this choice and the possible implications of the characteristic qualities of the environment.*

Response: As indicated in the introduction, conflicting results have been found in previous studies on the effect of visual feedback on pain-free range of motion. As suggested by the reviewer, a possible explanation for the different results between the two previous studies can be that different VR-environments were used (i.e., a countryside, park, mountain, church grounds, dining room and living room by Harvie et al.^4^, and different locations in one forest-environment by Kragting et al.^3^). In this study, we chose to use similar (indoor and outdoor) environments as used in the study of Harvie, to make the studies comparable and avoid bias. We included this consideration in lines 199-200.

Furthermore, a previous study that aimed to investigate the differential influence of multiple VR-environments on pain, reported that pain sensitivity was not modulated by context^5^. This information is used in the discussion (see line 377-378).

**Reviewer: # 2**

Comments to the Author
General comment - The aim of this study is clinically relevant and it will be of interest to clinicians. However, to be even more clinically and scientifically relevant, the results must be reinterpreted according to current guidelines in terms of p values.

Response: We agree with this reviewer’s remark. We added the confidence intervals in Table 2 and focused more on the effect sizes than on the statistical significance.

*1. Line 2 – “Are people with non-specific neck pain”?*

Response: We have incorporated this suggestion in the title.

*Abstract*

*2. Line 36 – Specify “people with non-specific neck pain” and add the comparison group (fear vs no fear).*

Response: We have specified the neck pain group (see line 39). The comparison group is described in lines 42-45.

*3. Line 37 – Specify the population (e.g., acute, subacute, chronic)? It would be nice to have this specification for the whole manuscript.*

Response: We have specified the population regarding the stage of the disorder (acute, subacute, chronic, long-lasting chronic) in Table 1 and added the IPQ 25-75% regarding ‘duration of neck pain’. In our opinion, this information is too detailed for an abstract. We decided not to include this specification (acute, subacute, chronic, long-lasting chronic) in the whole manuscript, as our previous study showed that the duration of neck pain had no impact on the effect of visual feedback manipulation on pain-free range of motion^3^.

*4. Line 39 – Replace “impact” with “effect” to be consistent.*

Response: Thank you for this comment. We replaced it (line 42).

*5. Line 44 – A p-value > 0.05 does not mean that there is no effect. Actually, the effect is η² = 0.044. This effect is really close to the other one (η² = 0.06) with a p-value < 0.05. I advise you to read the American Statistical Association statement about p-values. It is not recommended to focus on the “statistical significance” (p < 0.05) anymore.*

Response: We agree with the reviewer’s remark. We added the confidence intervals in Table 2 and focused more on the effect sizes, rather than on the statistical significance.

Introduction

*6. Line 56 – It may be interesting to add a definition of “non-specific neck pain”, because you included traumatic neck pain in the sample and this type of neck pain is not always included in the “non-specific neck pain” population.*

Response: We defined non-specific neck pain as ‘neck pain without a specific underlying pathology’^1^ (See line 61). A further specification of the characteristics of the participants is described in the selection criteria in the Methods section. (See lines 93-100).

*7. Line 58 – “that may be perceived as threatening”?*

Response: Thank you for this comment. We adjusted the sentence (line 63).

*8. Line 66 – Again, it is not right to state that there was no effect only based on p-values. In interpreting the results, we should include the exact p-value, the effect size, and its 95%CI. Furthermore, it should be better to show the effects found in that study and then compare them with the effect found in the other one. If there are differences in effect sizes, it can be very interesting to find some possible explanations.*

Response: In line with the general comment, the interpretation of the results of the studies we referred to in the introduction are based on the exact p-values, effect sizes and its 95%CI. In our opinion, it is unusual to include the explicit results of other studies in the introduction. A possible explanation for the different results between the two studies is given in the next paragraph (see lines 75-76).

*9. Lines 74-75 – “Individuals who are not fearful of movement may unlearn the association made between movement and pain, allowing pain to extinguish”. This is a strong hypothesis, because it says that pain can be extinguished only because of the unlearned association. However, not all people with non-specific neck pain have fear of movement, but they still suffer from pain.*

Response: We agree with the reviewer that in people with non-specific neck pain, there can be multiple reasons for the persistence of pain. In this study, we started from the hypothesis that pain may, partially, be a learned experience. We then propose a possible explanation for this hypothesis. To emphasize that it is an assumption, we have made the following adjustment ‘The underlying assumption is that individuals who are not fearful of movement may unlearn the association made between movement and pain, allowing pain to extinguish’ (see lines 80-81).

*10. Lines 78-79 - Maybe replace with "the aim for the current study was to evaluate whether the effect of visual feedback manipulation is greater in people with non-specific neck pain that are fearful of movement, compared to people with non-specific neck pain that are not»? Or something like that. However, in both cases, your hypothesis seems to be a superiority one (“more susceptible”). It will have an impact on the statistical hypothesis and testing used.*
Response: We agree with the reviewer that the objective of the study should have been formulated neutrally. We've adjusted this (see lines 84-86).

*Methods and materials*

General comment – The manuscript does not follow best practices for reporting. In addition, there is no registered protocol of the experiment.

Response: We followed the guidelines for reporting, however, we confirm that the protocol for the study was not published or registered.

General comment - You separated people with fear into two subgroups (TSK and FABQ-pa). I understand, but as you said earlier, these are two closely related constructs of fear. So why did you not combine them together into one group (“fearful people”)? And after that, it can be divided into two subgroups to see if there are some differences in the effect size*.*

Response: This comment of the reviewer made us reflect on our analyses. We formulated three different options how to modify our paper and sought advice from the Editor and reviewer (see Author Query 30-3-2023). Based on the advice received, we re-analysed our data (see comment 24).

*11. Line 84 –* *What was the sampling method used? Was it probabilistic (random) or non-probabilistic (e.g., convenience, snowballing)? It should be specified as it impacts the generalizability of the findings .*

Response: We added the sampling method used (see lines 92).

*12. Line 95 – “Based on an expected effect size of ηp² = 0.145 (i.e., 0.29/2)”. What is the basis for that? Why did you divide the effect by 2? If there is no specific reason, you should specified that it was arbitrary.*

Response: The design of the reference study (within comparisons)^4^ differed from the design of the current study (between-within), therefore we anticipated a smaller effect size. Indeed, the choice to divide the expected effect size by two was arbitrary. We specified this in the text (see lines 102-105).

*13. Line 110 – “motion sickness was evaluated”. Why? What information did it provide? Is it related to the purpose of the study?*

Response: We monitored motion sickness in this study. Motion sickness is a common side effect in VR, especially in people with neck pain and a possible barrier when considering implementing VR in clinical practice (see lines 119-121). Furthermore, in this study we increased the gain compared to other studies (i.e., manipulation of the visual feedback with 30% rather than 20%). In our opinion it is important to monitor this possible side effect, because it is a frequently mentioned phenomenon.

*14. Line 132 – Please add a reference for the cut-off score of FABQ-pa.*

Response: We added a reference for the cut-off score of the FABQ_pa_ (see line 141).

*15. Line 143 – I don’t understand why this outcome is assessed. I don’t think there is a link with the purpose of the study. Is motion sickness susceptible to influence the pain-free ROM or another outcome? If yes, it should be specified in the introduction.*

Response: Indeed, we have chosen not to include the evaluation of motion sickness in the objectives of the study. In our opinion this was a minor aim. An explanation for the choice made is given in comment 13.

*16. Line 184 – Please specify the method of randomization.*

Response: We specified the method of randomization in line 193-195.

*17. Line 186 – There are six environments. So, one environment per repetition for each condition? It is not clear.*

Response: To clarify the procedure we added ‘Hence, one environment was used per repetition for each condition’ to the sentence in line 198-199.

*18. Line 194 – “different”. Here you say "different" (equivalence or not), but the aim of the study was to evaluate whether the effect was greater in fearful subjects, to see if they are more susceptible (superiority or not). The hypotheses are not the same, as well as the type of p-value (one-sided or two-sided ).*

Response: As suggested by the reviewer in comment 10, we have reformulated our aim (see lines 84-86). This is in line with the formulation used in this section.

*19. Line 194 - It is stated that there are only 2 groups (with fear and without fear), however in the analyses there are 3 groups (without fear, fear with TSK, fear with FABQ-pa). It needs to be clarified.*

Response: In accordance with the advice, we performed 2 analyses with 2 subgroups per analysis (i.e., ‘no fear on both scales’ (TSK≤37 & FABQ-pa≤14) compared with ‘fearful for movement- kinesiophobia’ (TSK>37); and ‘no fear on both scales’ compared to ‘fear for physical activity’(FABQ-pa>14)). This is clarified in the text (see lines 206-211).

*20. Line 195 – Why is the absolute data analysis not included in the manuscript ?*

Response: As reported in the manuscript, we chose to report the relative data analysis in the main manuscript, because we wanted to anticipate expected differences between subjects (especially between people with and without fear of movement). Indeed, in the current sample the mean range of rotation differed between the people with and without fear of movement. To account for differences in the overall neck range of motion between subjects, for each participant, the data from the overstated and understated condition were transformed to a proportion of the mean range of rotation in the control condition (i.e., relative data). We have chosen not to include the absolute data-analysis in the main manuscript to avoid an overload of results, and thus confusion. Based on the reviewer's comment, we have decided to include the results of the absolute analyses in Table 2. We think this will support the clinician in interpreting the results.

*21. Line 202 - Thresholds are not clearly distinct, there are overlaps (e.g., 0.059 is small or medium ?).*

Response: Thank you for this comment. We made an adjustment (see lines 214-215).

*22. Line 205 – “two subgroups”. In total there are 3 subgroups. Maybe you want to say that there are 2 subgroups per analysis? If yes, please specify.*

Response: Thank you for this comment. We specified this (see line 217).

*Results*

*General comment – Results need to be reinterpreted according to the American Statistical Association statement on p-values. We should not use the “statistical significance” (p<0.05) to conclude if there is an effect or not. Results should be described with exact p-values, effect size, and its 95%CI. Adding the relative changes of range of motion would be great, as it allows readers to better understand the changes observed and to judge if they may be of clinical relevance (effect sizes in terms of η² might be difficult to interpret for clinicians).*

Results are described with exact p-values and effect sizes. Both the absolute and relative changes in range of motion (including their 95%CI) are added in Table 2. We agree with the reviewer that this will support readers to better understand the changes observed and to judge if they may be of clinical relevance.

*23. Line 218 – In the methods it is stated that 40 subjects were needed (according to the sample size calculation, 20 per group). However, 75 people participated. Why ?*

Response: The subgroups (fear/no fear) were not equally distributed. We continued to enrol participants until we could include about 20 people with fear of movement.

*24. Lines 221 to 223 – If I understand, you included people with fear of physical activity into the "no fear" group (compared to TSK), and you included people with kinesiophobia into the "no fear" group (compared to FABQ). If yes, I am not sure this is right because they are fearful in both cases. I don't think they can be considered as "no fearful". Including people with kinesiophobia or fear of physical activity in the “no fear” group may induce biases in the results as they are fearful in both cases. So, you compared “fearful” people with “fearful + no fearful” people .*

Response: We agree with the reviewer that a positive score on one of the two scales may indicate a form of fear, which may have biased the results. Classifying the people who scored positive on the TSK, but negative on the FABQ-pa, in the 'no-fear of physical activity group’ probably made the contrast between the two groups smaller. The same applies to the analysis based on the TSK. In accordance with the advice from the reviewer (see email dd. 06-04-2023), we reanalysed the data and only included people who scored negative on both scales (TSK≤37 &FABQ-pa≤ 14) in the no fear group. We then also added an exploratory analysis in which people who were negative on both scales (N=46) were compared to participants who scored positive on both scales (N=10). The effect size is then twice as large (p=0.007, ղ2=0.112), confirming the impact of fear.

*25. Line 223 – “10 participants had fear according to both questionnaires”. So, these participants were included twice in the analyses? Once for TSK and once for FABQ-pa ?*

Response: as mentioned in our response in comment 19 & 24 we performed 2 separate analyses with 2 subgroups per analysis (i.e., ‘no fear on both scales’ (TSK≤37 & FABQ-pa≤14) compared with ‘fearful for movement- kinesiophobia’ (TSK>37); and ‘no fear on both scales’ compared to ‘fear for physical activity’(FABQ-pa>14)). So indeed, most participants were included twice in the analyses. As mentioned in comment 24 we also added an exploratory analysis (to enlarge the contrasts between the subgroups) in which people who were negative on both scales (N=46) were compared to participants who scored positive on both scales (N=10).

*26. Line 225 – “no differences”. Based on what? If you refer to the p-values, please indicate the exact p-value. However, it would be interesting to add the values of age and duration of neck pain for both groups. As mentioned earlier, the p-value alone is not sufficient to state that there is a difference or not .*

Response: To compare the participants characteristics between the subgroups, we performed statistical tests (independent sample t-tests or (in case of non-normality of the data) non-parametric Mann-Whitney tests). We chose to report values of age, duration, pain intensity, disability, kinesiophobia, fear of physical activity and ROM, including the results of the statistical tests, in Table 1 and not in the text, as these results do not answer the main research question and we intended to avoid duplication. Therefore, we suggest referring to the table for these data (as done in the original version)

*27. Lines 226-227 – “**more disabled, scored higher on pain intensity and was more limited in the pain-free range of rotation”. Based on what? If you refer to the p-values, please indicate the exact p-value for each comparison. However, it would be interesting to add the values of pain intensity, disability, and ROM for both groups. As mentioned earlier, the p-value alone is not sufficient to state that there is a difference or not.*

Response: We specified in the text that ‘Table 1 provides an overview of the participant characteristics per group including the results from the comparisons between subgroups’ (see line 239-240).

*28. Line 230 – What about people with fear of physical activity ?*

Response: We added the result regarding mean ROM in people with fear of physical activity (see line 243).

*29. Line 243 – “Numeric” rather than “Nummeric”.*

Response: Thank you for this comment. We made an adjustment (see line 254).

*30. Line 255 – It is not recommended to use the “statistical significance” anymore. Please read the American Statistical Association statement on p-values. It would be great to show the relative change in range of motion and its 95%CI where appropriate. In addition of the effect size, it gives a better idea of the effect (it is well done in the S3 appendix). Interpreting these effects as clinically relevant or not would also be a good idea.*

Response: The exact p-values and effect sizes are described and the effect sizes are interpreted. The 95% CI are expressed in Figure 2 and Table 2. The absolute values (mean ROM and 95%CI) have been added in Table 2. This has informative value for the clinician.

*31. Lines 263 to 266 – Do not use statistical significance. For example, you could say that “differences in range of motion between the overstated gain and the control condition (p=0.051, ղ2=0.052), and between the understated gain and the control condition (p=0.077, ղ2=0.044) were a bit smaller than the effect found between the understated and the overstated conditions”. Or something like that, to focus not on statistical significance but on effect sizes. Adding a 95%CI for effect sizes would also be good.*

Response: As previously mentioned: results are described with exact p-values and effect sizes. Effect sizes are interpreted, and both the absolute as relative changes in range of motion (including their 95%CI) are added in Table 2.

*32. Line 272 – “conflicting” rather than “conflciting”. Why is it “conflicting”? Is it because the p-value is superior to 0.05?*

Response: We agree with the reviewer that the result of our prior analyses were not conflicting. We only found a small effect in the TSK group. The results of the recent analyses are in line with each other.

*33. Line 280 – “Median” rather than “mean”?*

Response: Thank you for this comment. Following Field (3th ed, p. 580)^6^, we have to report the test statistic, degrees of freedom and its significance. We made an adjustment (see line 315).

*34. Line 283 – You indicated 75 participants before, now it is 76. Please correct.*

Response: Thank you for this comment. We made an adjustment (see line 318).

*35. Line 287 – I still don’t understand why this outcome is evaluated and its usefulness in this study.*

Response: See response in comment 13

*Discussion*

*36. Line 303 – Replace “pain-related” by “pain-free” to be consistent.*

Response: Thank you for this comment. We checked the manuscript for the use of “pain-free”

*37. Lines 306-307 – Can these factors play a role in the effects observed? If yes, it should be discussed. You do not mention these factors as potential confounders in the differences in the effects observed.*

Response: It was expected that the differences in pain-free range of motion between the groups with and without fear of movement would influence the scores in the absolute data-analyses. Therefore the relative range of motion scores were used in the main analyses. This is already explained in the text (lines 187-191). It was not expected that the scores for pain and dysfunction were potential confounders, as a within design is used to test the effect of feedback manipulation on pain-free range of motion and the scores for pain and functioning were the same for each participant in the three gain conditions.

*38. Lines 321 to 325 – First, the absolute range of motion scores are detailed in the S3 Appendix, however I am not sure that readers will look at it. These scores are discussed here without any specific reason, as you mentioned earlier in the manuscript that you would use the relative data (to account for differences in the overall neck range of motion). Why do you discuss the absolute changes and not the relative ones? In the manuscript, you only show the analyses for the relative data, but in the discussion, you only mention the absolute data . It is not clear. Second, these differences in absolute range of motion are on the order of 4-5 degrees for the total range of motion in rotation. Is it clinically relevant? I think it would be good to specify the clinical relevance of these findings.*

Response: The absolute range of motion scores are easier to interpret, while the relative scores have no clear meaning for clinicians. However, we agree with the reviewer that it is strange to discuss the absolute data while in the rest of the manuscript the relative data were described. Therefore, this part is moved to the appendix. To make the relative data easier to interpret for clinicians, the change in pain-free range of motion is expressed in a percentage increase or decrease in range of motion.

*39. Line 338 – Why is it convincing? What is your basis for that?*

Response: This part of the text has been omitted in the revised version of the discussion

*40. Line 343 - Again, "no effect" means that the effect size equals 0. Was it the case? Or just a p-value > 0.05?*

Response: In this case we think we can say there is ‘no effect’, as the effect size is small, and the p-value high (p=0.133, ղ_p_^2^=0.031).

*41. Lines 367-368 – The conclusion is a bit too ambitious. I advise you to interpret according to the results. Maybe something like "In people with non-specific neck pain, those with fear of movement may be more susceptible for the effect of visual feedback manipulation than people without fear of movement. Because the effect sizes are small to medium and because of the limitations mentioned, the results should be interpreted with caution”. Results should also be interpreted according to the sample characteristics (more females, middle-aged people, duration of neck pain, mean pain intensity, disability). Please revise it in the abstract too.*

Response: We believe that the first statement (‘This experiment supports the view that pain-free ROM can be influenced by visual perception of the amount of rotation (i.e., the main effect of gain, independent of the presence of fear)’) can be well substantiated by the results (TSK: p<0.001, ղ_p_^2^=0.158; FABQ_pa_: p<0.001, ղ_p_^2^=0.195) -> large effect sizes. Given the medium effect sizes in both the analyses regarding the influence of fear (i.e., the interaction effect between fear of movement and visual feedback manipulation), we think that the interpretation that ‘People with fear of movement seem to be more susceptible for the effect of visual feedback manipulation than people without fear of movement’, is sufficiently consistent with the results (see lines 410-413).

*S3 Appendix*

*42. Line 4 - The Kolmogorov-Smirnov test is used to assess normality. However, in the manuscript it is stated that normality is assessed via Q-Q plots and histograms. Why is it different here?*

Response: We used the Q-Q plots, the histograms and the KS test to consider if the data were sufficiently normally distributed, so we have adjusted this in the text.

*43. Lines 13 to 17 – Interaction effect was present in both cases, however it was no “significant” based on the “p<0.05” threshold. Please see the American Statistical Association statement about p-values. These results need to be reinterpreted.*

Response: In accordance with the advice (see comment 24 and 25) we also re-analysed the data using the absolute range of motion scores. We performed 2 analyses with 2 subgroups per analysis (i.e., ‘no fear on both scales’ (TSK≤37 & FABQ-pa≤14) compared with ‘fearful for movement- kinesiophobia’ (TSK>37); and ‘no fear on both scales’ compared to ‘fear for physical activity’(FABQ-pa>14)). The analyses and results are reinterpreted, following the American Statistical Association statement. The confidence intervals are expressed in Table 2 and S3 Figure.

*44. Lines 13 to 30 – Are these mean changes clinically relevant ?*

Response: The mean changes in the ‘no fear’ group do not seem to be clinically relevant (as also discussed in the main manuscript (lines 390-398), while the changes in pain-free range of motion in the ‘fear of physical activity group’ and the ‘kinesiophobia’ group, might be clinically relevant. This is explained in lines 401-407.

References

1.	Tsakitzidis G, Remmen R, Peremans L, et al. Non-specific neck pain: diagnosis and treatment. *Good Clinical Practice (GCP) KCE Reports C.* 2009;119.

2.	Bier JD, Scholten-Peeters, G.G.M., Staal, J.B., Pool, J., Tulder, M. van, Beekman, E., Meerhoff, G.M. Knoop, J., Verhagen, A.P., . KNGF richtlijn nekpijn. In. *Praktijkrichtlijn*. Amersfoort: KNGF; 2016.

3.	Kragting M, Schuiling SF, Voogt L, Pool-Goudzwaard AL, Coppieters MW. Using Visual Feedback Manipulation in Virtual Reality to Influence Pain-Free Range of Motion in People with Nonspecific Neck Pain. *Pain Pract.* 2020.

4.	Harvie DS, Broecker M, Smith RT, Meulders A, Madden VJ, Moseley GL. Bogus visual feedback alters onset of movement-evoked pain in people with neck pain. *Psychol Sci.* 2015;26(4):385-392.

5.	Smith A, Carlow K, Biddulph T, Murray B, Paton M, Harvie DS. Contextual modulation of pain sensitivity utilising virtual environments. *Br J Pain.* 2017;11(2):71-80.

6.	Field A. *Discovering Statistics Using SPSS.* Third ed. London: Sage Publications Ltd; 2009.
