# Supplementary material for: Visual feedback manipulation in virtual reality to influence pain-free range of motion. Are people with non-specific neck pain who are fearful of movement more susceptible?
Source: PLoS One. 2023 Jul 5;18(7):e0287907. doi: 10.1371/journal.pone.0287907 (PMC10321611; doi:10.1371/journal.pone.0287907)
Supplement: Attachment — Submitted filename: Response to Reviewers_VR_Fear_revision2_DEF.docx [file pone.0287907.s007.docx]

| datum | ons kenmerk | uw brief van | uw kenmerk |
| --- | --- | --- | --- |
| 8 June 2023 |  |  |  |
|  |  |  |  |

To:	Prof. Mariella Pazzaglia

Academic Editor PlosOne

Article number: PONE-D-22-28987.

*Visual feedback manipulation in virtual reality to influence pain-free range of motion. Are people with pain who are fearful of movement more susceptible?’*

Dear Prof. Mariella Pazzaglia,

Thank you for giving us the opportunity to conduct the minor revisions requested for the article mentioned above.

We would like to thank you and the reviewer for the helpful comments, positive feedback and clear suggestions to further improve the quality of the manuscript. We have addressed each of the points raised. In the response letter, the reviewer comments are printed in black and italic, followed by our response in regular blue font.

All authors read and approved the revised manuscript. We look forward to your response.

On behalf of all the authors,

Yours sincerely,

Prof. Michel Coppieters

**Author response letter**

**Journal Requirements:**

Comments to the Author
*Please review your reference list to ensure that it is complete and correct. If you have cited papers that have been retracted, please include the rationale for doing so in the manuscript text, or remove these references and replace them with relevant current references. Any changes to the reference list should be mentioned in the rebuttal letter that accompanies your revised manuscript. If you need to cite a retracted article, indicate the article’s retracted status in the References list and also include a citation and full reference for the retraction notice.*

Response: We reviewed our reference list to ensure that it is complete and correct.

**Reviewer: # 2**

Comments to the Author

Introduction

1. *Lines 80-81 – The authors said “to emphasize that it is an assumption, we have made the following adjustment ‘The underlying assumption is that individuals who are not fearful of movement may unlearn the association made between movement and pain, allowing pain to extinguish’”, in relation to the comment number 9 (first revision). It's clearer that this is a hypothesis. However, my comment was more about the "allowing pain to extinguish" part. By saying this, the authors are assuming that the association between movement and pain is the only factor that determines the presence of pain. I'm not so sure about that. I think pain can be reduced, but not necessarily extinguished. I would suggest being more nuanced in the sentence and saying, for example, "allowing pain to be reduced".*

Response: We followed the suggestion and changed ‘extinguished’ to ‘reduced’ (lines 82-83)

Methods and materials

*2. General comment – The authors have confirmed that they have followed reporting guidelines, but these are not mentioned in the manuscript. Please specify which reporting guidelines were followed in this manuscript.*

Response: We added the reporting guideline to the manuscript (lines 111-112). Furthermore, we specified the study design in the Abstract (line 39) and we added the recruitment period to the Methods (line 92).

*3. Line 91 – I think that the first part of the sentence should be placed at the beginning of the results. The number of subjects is also written at line 238, but with different characteristics in brackets. It should be better to combine these two sentences in one (only the number of subjects and their characteristics) to avoid repetition.*

Response: We have followed this suggestion and removed the duplication (lines 242-243)

*4. Lines 119-121 – “Since motion sickness is a common side effect in VR (17), especially in people with neck pain (18), and a possible barrier when considering implementing VR in clinical practice, it was evaluated after the VR-experiment using the short version of the Misery Scale (sMISC)”.*

Response: Thank you for your suggestion. Given the length of the sentence, we chose to split the sentence (lines 121-123)

*5.* Line 131 – “(FABQpa)” rather than “(FABQpa))”.

Response: Corrected as suggested (line 133)

*6* *Line 132 – “Cut-off” rather than “cut-of”.*

Response: Corrected as suggested (line 134)

*7. Line 212 – To be as complete and transparent as possible, I would suggest specifying that you will present results using exact p-values, the effect estimate and its 95%CI. Avoiding the use of the threshold "p<0.05" is not yet widespread in musculoskeletal research, so readers may be confused and wonder why you don't specify whether the results are statistically significant or not, if this is not stated here.*

Response: We agree with the reviewer’s suggestion. We specified this in the manuscript (lines 224-225).

Results

*8* *Line 238 – My comment was: “In the methods it is stated that 40 subjects were needed (according to the sample size calculation, 20 per group). However, 75 people participated. Why?”. Authors responded: “The subgroups (fear/no fear) were not equally distributed. We continued to enroll participants until we could include about 20 people with fear of movement.”. I understand, but why didn't you keep the first 20 subjects with no fear of movement and only continue to enroll subjects with fear of movement? In any case, it's already been done. So, I suggest clarifying in the manuscript what you mentioned above, so that the reader is not confused about the difference between the sample size calculation and the final sample size.*

Response: We followed the reviewer’s comment and incorporated this suggestion (lines 244-246)

*9. Lines 244-245 – It says "In both groups" at the beginning of the sentence, but don't you mean "In both analyses"? I think it is not clear enough, because you have two fearful subgroups.*

Response: Thank you for identifying this. Changed as suggested. (lines 250)

*10. Line 250 – Add “pain-free” before “range of rotation” to be consistent.*

Response: Changed as suggested (line 256) and throughout the manuscript.

*11. Line 251 – Add “degrees” after “… with fear of physical activity 106.9 (38.4)”, to be consistent.*

*Response:* Changed as suggested *(line 257)*

*12. Table 1 – I don't think it's necessary to specify both sexes. In general, we only specify one sex (female or male), because if we know the numbers for one, we indirectly know the numbers for the other.*

Response: Changed as suggested and incorporated this in Table 1.

*13. Line 265 – If you use a specific threshold for significance for these tests, you should specify which one you are using.*

Response: Changed as suggested - We added whether we used t-test or Mann-Whitney U tests in Table 1.

*14. Lines 281-285 – At the beginning of the sentence (starting with “Contrasts…”) you compare pain free range of motion between the understated condition and the control condition, in fearful people, but in the next part of the sentence it is less clear. Do you talk about overstated versus control condition in fearful and non-fearful people, or do you compare fearful vs non fearful people in both conditions?*

Response: The contrasts reveal the differences between people with and without fear of movement in the understated condition compared to the control condition, and the differences between people with and without fear of movement in the overstated condition compared to the control condition. We modified the text to make this clear (lines 288-293).

*15. Line 282 – If you avoid using a specific threshold for statistical significance, it is best to avoid terms like “significantly”.*

Response: We agree with the reviewer. Changed as suggested. (line 289)

*16.* *Lines 287-289 – The following part of the sentence is not clear for me: “decreased by 4.5%, 95%CI [-9.0%, 0.0%] (TSK) respectively 6.5%, 95%CI [-9.9%, -3.1%] (FABQpa) in the overstated condition.”. Could you rewrite it more clearly?*

Response: We modified the text and wrote this part more clearly (see lines 293-300).

*17. Line 289 – “This direction of the effect of visual feedback was as expected”. For me, this should be placed in the discussion.*

Response: We agree and adjusted the manuscript accordingly (lines 391-393).

*18. Lines 289-290 – I had to reread the previous sentences to understand what the "overall effect" was. I think it would be a good idea to add in brackets what exactly it is.*

Response: Changed as suggested: we added “(i.e., the difference in pain-free range of motion between the understated and the overstated condition)” (lines 300-303)

*19. Lines 324-325 – Same comment as for lines 289-290.*

Response: Changed as above (lines 337-339)

*20. Lines 340-342 – For completeness and consistency, I would add the number of subjects with each type of symptom, in addition to the percentage (which is done in the previous section).*

Response: Unlike for the ‘previous section’, these percentages are already mentioned in Table 3. We prefer not to follow the suggestion of the Reviewer to avoid duplication.

Discussion

*21. Line 353 – What do you mean by “susceptibility to visual feedback manipulation”? Maybe “susceptibility for the effect of visual feedback manipulation”?*

Response: We have incorporated this suggestion (line 366).

*22. Lines 388-389 – My comment was: “Again, "no effect" means that the effect size equals 0. Was it the case? Or just a p-value > 0.05?”. Authors responded: “In this case we think we can say there is ‘no effect’, as the effect size is small, and the p-value high (p=0.133, ղp2=0.031)”. I don’t agree. To be consistent with the rest of the manuscript, authors should replace “no effect” with “small effect”.*

Response: Changed as suggested (line 403).

*23.* Line 390 – Add “of” after “fear”.

Response: Changed as suggested (line 406).

*24. Lines 408-421 – If you want to compare the fearful and non-fearful groups in the same way, you need to use the same relative changes. For non-fearful subjects, you're talking only about the change between the overstated condition and the control condition, whereas for fearful subjects, you're talking about the overall relative change. So, there's a big difference between the two groups.*

Response: We agree with the reviewer that when comparing the fearful and the non-fearful groups in the same way, we have to compare the relative changes in the same conditions. We therefore compared the overall relative change in both groups in the revised version of the manuscript (lines 424-430).

*25. Lines 426-427 – I would add “neck” before “pain-free range of motion” and specify that it is about rotation only. Sometimes you write “pain-free range of motion”, sometimes “cervical pain-free range of motion” and sometimes “neck pain-free range of motion”. Try to be consistent so that things are always written in the same way. In addition, I would clarify the population again. For example, the new sentence would be: “This experiment supports the view that neck pain-free range of motion in rotation can be influenced by visual perception of the amount of rotation, in people with non-specific neck pain”.*

Response: Changed as suggested. We preferred to use ‘cervical’ instead of ‘neck’. (lines 444-445)
